# Metastasis-associated protein 1 is an upstream regulator of *DNMT3a* and stimulator of insulin-growth factor binding protein-3 in breast cancer

**DOI:** 10.1038/srep44225

**Published:** 2017-04-10

**Authors:** S. Deivendran, Hezlin Marzook, T. R. Santhoshkumar, Rakesh Kumar, M. Radhakrishna Pillai

**Affiliations:** 1 Cancer Research Program, Rajiv Gandhi Centre for Biotechnology, Thiruvananthapuram 695014, India; 2 Department of Biochemistry and Molecular Medicine, School of Medicine and Health Sciences, George Washington University, Washington, DC 20037, USA

## Abstract

Despite a recognized role of DNA methyltransferase 3a (DNMT3a) in human cancer, the nature of its upstream regulator(s) and relationship with the master chromatin remodeling factor MTA1, continues to be poorly understood. Here, we found an inverse relationship between the levels of MTA1 and DNMT3a in human cancer and that high levels of MTA1 in combination of low DNMT3a status correlates well with poor survival of breast cancer patients. We discovered that MTA1 represses *DNMT3a* expression via HDAC1/YY1 transcription factor complex. Because IGFBP3 is an established target of DNMT3a, we investigated the effect of MTA1 upon IGFBP3 expression, and found a coactivator role of MTA1/c-Jun/Pol II coactivator complex upon the *IGFBP3* transcription. In addition, MTA1 overexpression correlates well with low levels of DNMT3a which, in turn also correlates with a high IGFBP3 status in breast cancer patients and predicts a poor clinical outcome for breast cancer patients. These findings suggest that MTA1 could regulate the expression of IGFBP3 in both DNMT3a-dependent and -independent manner. Together findings presented here recognize an inherent role of MTA1 as a modifier of DNMT3a and IGFBP3 expression, and consequently, the role of MTA1-DNMT3a-IGFBP3 axis in breast cancer progression.

Aberrant epigenetic changes and resulting dysregulated gene expression programs represent an important underlying mechanism of hyperactivation of cellular pathways that drive the process of tumor progression. The outcome of gene transcription machinery depends on a coordinated participation of regulatory and feed-back mechanisms and state of chromatin remodeling on target genes. The core of epigenomic control of gene expression is regulatory inter-plays among histones, nuclear factors, chromatin remodeling factors, and nucleosome landscape on the target gene chromatin, and target DNA modifications including, methylation. It is generally believed that any combination of dysfunction of these events and/or molecules could contribute to an aberrant gene expression during cancer progression.

One of the crucial chromatin remodeling factors in eukaryotic cells is the metastasis-associated protein 1 (MTA1) - one of the most upregulated oncogenes in human cancer that contributes to cancer progression and metastasis^1^. The MTA1 master coregulator acts as an effector for a wide variety of upstream signals and interacting oncogenes^2^. At the cellular level, MTA1 overexpression in cancer cells regulates a variety of pathways that contribute to the processes leading to invasion, epithelial-to-mesenchymal transition, survival, and metastasis. In-spite of significant advances in our understanding of the mechanisms of MTA1 overexpression associated cancer progression, the nature of molecular relationship between the MTA1 chromatin remodeling factor and DNA methyltransferases remains poorly understood.

A critical regulatory DNA modification is through methylation which acts as a switch for gene expression via interacting with the chromatin remodeling complexes^3^. The process of DNA methylation is influenced by DNA methyltransferases enzymes, including DNMT1, DNMT3a, and DNMT3b. In general, DNMT1 is a maintenance methyltransferase while DNMT3a and DNMT3b contribute to de-novo methylation^4^. In addition to downstream events, dysregulation of upstream regulators of DNMT expression also contribute to a disorderly regulation of genes with roles in events underlying the process of cancer progression^5^. DNMT3a has been shown to be associated with establishment of methylation pattern during embryonic germ cell development^6^. DNMT3a overexpression in certain cellular systems correlates well with the process of tumorigenesis in breast, liver, leukemia, colon and prostate cancers^7^. Interestingly, inhibition of DNMT3a leads to upregulation of immune response genes in melanoma^8^ and tumor suppressor PTEN in hepatocellular carcinoma cells^9^. However, DNMT3a depletion promotes tumor cell progression, cell adhesion, angiogenesis in lung cancer cells^10^, suggesting an unusual tumor suppressor role of DNMT3a in some cell-types. It is noteworthy to mention that the genetic depletion of *Dnmt3a* does not lead to a global change the status of net DNA methylation^11^, but limits alterations of methylation and expression patterns of specific target genes^12^. These observations suggest that DNMT3a remains a least-understood family member.

Here we investigated the role of MTA1 in the regulation of DNMT3a expression in cancer cells. We found that the levels of MTA1 and DNMT3a are inversely correlate in human cancer at-large, and that low DNMT3a in combination of high MTA1 expression predicts a poor clinical outcome in breast cancer patients. The underlying mechanism includes MTA1 repression of *DNMT3a*-transcription through recruitment of the MTA1/HDAC1/YY1 co-repressor complex onto the *DNMT3a* promoter chromatin. Interestingly, we also noticed that MTA1 stimulates expression of IGFBP3 - a molecule known to confer growth and survival advantages^13^,^14^ as well as a target of DNMT3a^15^,^16^. We found that MTA1 stimulates the expression of IGFBP3 by recruiting the MTA1/c-Jun/Pol II coactivator complex onto IGFBP3 promoter. Interestingly, low levels of DNMT3a correlates well with a high IGFBP3 expression and associate with a poor distant metastasis-free survival of breast cancer patients. We found that increased levels of MTA1 correlate well with an elevated level of IGFBP3 as well as low DNMT3a and this may result in poor prognosis of breast cancer patients. Since DNMT3a is known to negatively regulate IGFBP3, these findings suggest that MTA1 status may also regulate the expression of IGFBP3 through both DNMT3a-dependent and -independent pathways. In brief, findings presented here identified an upstream regulatory role of MTA1 coregulator in controlling the expression of DNMT3a and IGFBP3 in cancer cells and possibly modifying the biology of breast cancer progression.

## Results

**Figure 1:**
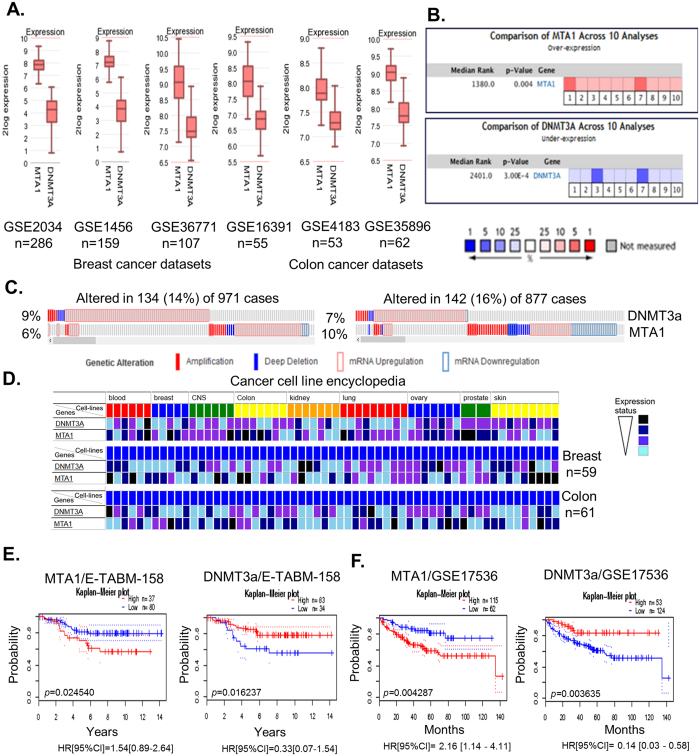
MTA1 levels negatively correlate with DNMT3a status in human cancer. (**A**) Levels of MTA1 and DNMT3a mRNAs in four breast and two colon cancer datasets as analyzed using R2: Genomics Analysis and Visualization Platform, and values are presented on a log2 scale. (**B**) levels of MTA1 and DNMT3a in cancer cells when compared to the normal cells in Oncomine Cancer Profiling Database. Box’s detail: 1, 10, gastric^39^,^40^; 2, anaplastic oligodendroglioma^41^; 3, renal cell carcinoma^42^, 4–5, Ovarian cell carcinoma^43^; 6–7, Skin^44^,^45^ and 8–9, colon cancers^46^ TCGA Colorectal, 2011]. The p-value is provided for the medium-rank analysis. (**C**) cBioPortal showing the genetic alterations in the DNMT3a and MTA1 in 971 patients obtained from TCGA-Breast cancer dataset; in 877 patients from Cancer cell line encyclopedia; each bar indicates the individual cases; % on the left indicates the percentage of cases altered in MTA1 and DNMT3a. (**D**) Panel 1: DNMT3a and MTA1 mRNAs in blood, breast, CNS, colon, kidney, lung, ovary, prostate and skin cancer cell lines in NCI-60; Panel 2, DNMT3a and MTA1 mRNAs in 59 breast cancer cell lines; and Panel 3, DNMT3a and MTA1 mRNAs in 61 colon cancer cells analyzed through CAncerREsource2 database. (**E**) Kaplan-Meier plot of disease specific survival of 117 breast cancer patients stratified by high (red) and low (blue) in the dataset E-TABM-158. (**F**) Kaplan-Meier plot of disease specific survival of 177 colon cancer patients stratified by high (red) and low (blue) analyzed through PrognoScan database. Dotted lines, 95% confidence intervals for each group.

To explore the nature of relationship between the status of MTA1 and DNMT3a in human cancer, we first interrogated the available breast and colon microarray public datasets for the levels of DNMT3a and MTA1. We noticed an inverse relationship between the levels of MTA1 and DNMT3a mRNAs in all data sets analyzed (Fig. 1A). As an example, MTA1 upregulation in breast tumors was accompanied by more than 3-fold downregulation of DNMT3a mRNA (Fig. S1, ref. 17). The noted inverse relationship between the levels of MTA1 and DNMT3a mRNAs was not limited to human breast or colon cancer but true for cancer, at-large, in general, when compared with the corresponding normal tissues using Oncomine dataset (Fig. 1B). For example, MTA1 and DNMT3a mRNA levels are significantly upregulated (*p* = *0.004*) and downregulated (*p* = *3.00E-4*) respectively, in gastric, renal, ovarian, skin and colon cancers when compared to the normal tissues (Fig. S2). Like Oncomine datasets, we also found an inverse relationship between the status of MTA1 and DNMT3a mRNAs in 971 cases of breast cancers from the TCGA dataset analyzed using cBioPortal tools (Fig. 1C). Next, we examined the status of MTA1 and DNMT3a in 877 cases from the Cancer Cell Line Encyclopedia using cBioPortal database. Once again, we found a remarkable reverse relationship between MTA1 upregulation and DNMT3a downregulation in most cancer types with a low degree of exception to this relationship (Fig. 1D). We also noticed an inverse relationship between the levels of MTA1 and DNMT3a in NCI-60 Panel cell lines as well as in 59 breast and 61 colon cancer cell lines (Fig. 1D).

To understand the significance of the noted inverse expression of MTA1 and DNMT3a, we performed survival analysis using PrognoScan^18^ tools. We found that MTA1 overexpression in breast tumors (n = 37) associates (*P* = *0.024540*) with a poor disease-specific survival (HR[95% CI] = 1.54[0.89–2.64]) of breast cancer patients (n = 37). Similarly, DNMT3a downregulation in breast tumors (n = 34) associates (*p* = *0.016237*) with a poor disease-specific survival (HR[95% CI] = 0.33[0.07–1.54]) (Fig. 1E). High levels of MTA1 (n = 115) and low levels of DNMT3a (n = 124) in colon cancer also results in a decreased probability of disease-specific survival having hazard ratio [95% CI] = 2.16 [1.14–4.11] for MTA1 and hazard ratio [95% CI] = 0.14 [0.03–0.58] for DNMT3a with statistical significance of *p* = *0.004287* and *p* = *0.003635*, respectively (Fig. 1F). Extension to this analysis to other cancer-types such as brain, lung, ovarian, and bladder also reinforced the notion that high MTA1 and low DNMT3a combination associates with an overall poor patient survival (Fig. S1B). These results suggest that the newly recognized inverse relationship between levels of MTA1 and DNMT3a might be one of the variables that could contribute to a poor prognosis of cancer patients.

### MTA1 is an upstream regulator of DNMT3a

**Figure 2:**
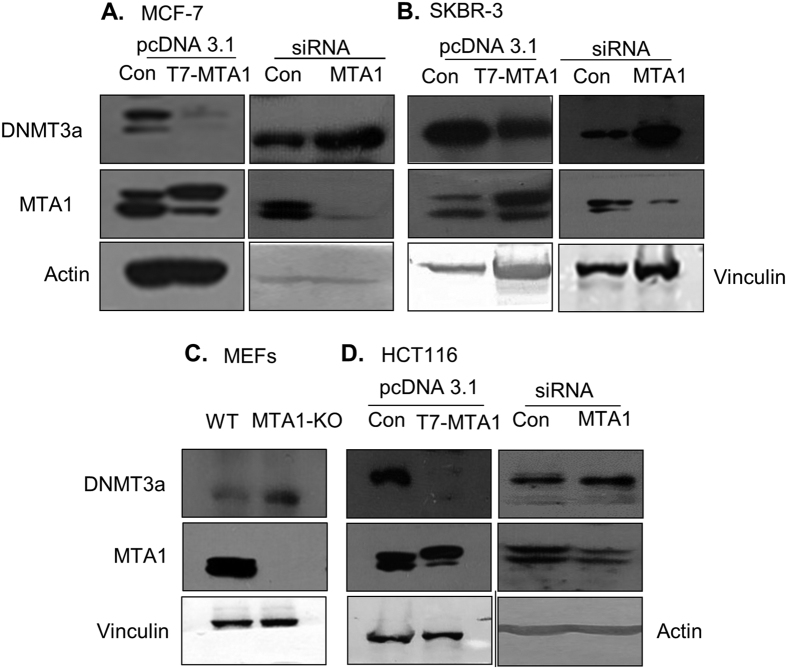
MTA1 regulates DNMT3a expression. (**A**) Western blot showing the levels of DNMT3a upon overexpression and silencing of MTA1 in MCF-7 cells. β-actin is showed as a control. (**B**) Western blot showing the levels of DNMT3a upon overexpression and silencing MTA1 along with the control in SKBR-3 cells. Vinculin is showed as a control. (**C**) Western blot showing the endogenous levels of DNMT3a and MTA1 and Vinculin in the wild type and MTA1^−/−^ MEFs. Vinculin is used as a control. (**D**) Western blot showing changes in the levels of DNMT3a upon overexpression or silencing MTA1 in HCT116 cells. β-actin and vinculin serve as loading controls, respectively.

Because of the significant clinical implications of the noted inverse MTA1-DNMT3a relationship, we next investigated the mechanism of MTA1 regulation of DNMT3a expression in cancer cells. To experimentally validate the noticed, presumed negative regulation of DNMT3a expression by MTA1, we selected breast cancer model system for subsequent studies. First, we examined the effect of overexpression or depletion of MTA1 on the status of DNMT3a in breast cancer cells. We found that MTA1 overexpression results in a marked reduction in the level of DNMT3a, while MTA1 silencing leads to an increased DNMT3a expression in breast cancer MCF-7 and SKBR-3 cells (Fig. 2A,B). Consistent with these results, we found that the genetic depletion of MTA1 in murine embryonic fibroblasts (MEF) accompanies with DNMT3a upregulation as compared to the level in the wild-type MEFs (Fig. 2C). Similar to breast cancer cells, MTA1 silencing or overexpression in HCT116 colon cancer cells also leads to upregulation or downregulation of DNMT3a, respectively (Fig. 2D).

**Figure 3:**
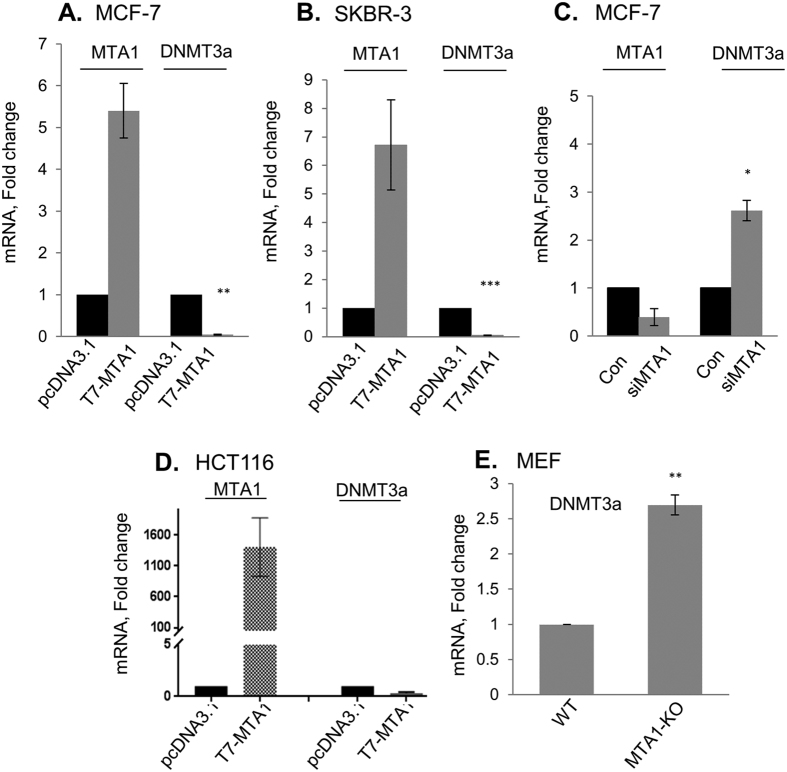
MTA1 regulates DNMT3a mRNA. (**A,B**) qPCR analysis of MTA1 and DNMT3a mRNA in MCF-7 cells and SKBR-3 cells upon overexpressing MTA1 as compared with the control vector. Results are presented as fold change after normalizing with β-actin and 18 S rRNA, respectively. (**C**) qPCR analysis of MTA1 and DNMT3a mRNA in MCF-7 upon MTA1 silencing as compared with the control vector. (**D**) qPCR analysis of MTA1 and DNMT3a mRNA in HCT116 by overexpressing MTA1 compared with the vector control. Results are presented as fold change after normalizing with β-actin. (**E**) qPCR analysis of DNMT3a in MTA1^+/+^ and MEF MTA1^−/−^ MEFs. Results are presented as fold change after normalizing with β-actin. (*p < 0.01, **p < 0.001, ***p <* 0.0001*)

The noticed modulation of DNMT3a expression by MTA1 was also reflected at the level of mRNA as MTA1 overexpression downregulates the levels of DNMT3a mRNA in MCF-7 (Fig. 3A) and SKBR-3 cells (Fig. 3B) significantly (*p* < 0.001); conversely, MTA1 knockdown leads to an increased level of DNMT3a mRNA in MCF-7 cells (*p* < 0.01) (Fig. 3C); marked reduction in DNMT3a mRNA levels were seen when MTA1 is overexpressed in HCT116 cells (Fig. 3D). MTA1-depletion in MTA1-KO MEFs leads to an increased significant level of DNMT3a mRNA (*p* < 0.01) as compared to wild-type MEF (Fig. 3E). In brief, these findings revealed that MTA1 is an upstream regulator of DNMT3a expression.

### MTA1 represses *DNMT3a* transcription

**Figure 4:**
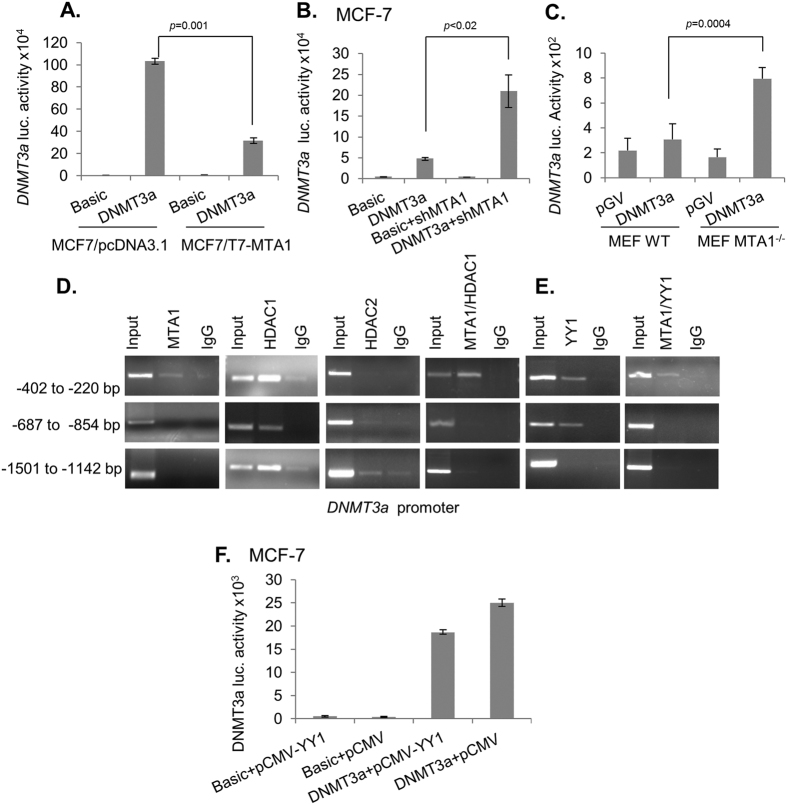
MTA1 represses *DNMT3a* transcription. (**A**) *DNMT3a* promoter activity in MCF-7/pcDNA3.1 and in MCF-7/T7-MTA1 cells. (**B**) *DNMT3a* promoter activity in MCF-7 and in MTA1-silenced MCF-7 cells. MCF-7 cells were transfected with pGL3-DNMT3a along with siMTA1 and *DNMT3a*-promo-Luc activity was measured. (**C**) *DNMT3a* promoter activity in MTA1^+/+^ and MTA1^−/−^ MEFs. (**D**,**E**) Recruitment of MTA1 or HDACs or YYI or MTA1/HDAC1 or MTA1/YY1 complexes onto the *DNMT3a* promoter in MCF-7 cells as analyzed by ChIP or sequential ChIP (**F**). The effect of YY1 on *DNMT3a-*promoter activity was measured in MCF-7 cells. Results were presented in terms of relative luciferase activity and the values represent the mean of ± s.d. from three independent transfection experiments.

To study the mechanism of MTA1 modulation of DNMT3a expression, we next measured the transcription of *DNMT3a* using a *DNMT3a-*prom-luc reporter system^19^ in MCF-7 cells under conditions of with or without MTA1 overexpression. We noticed a substantial repression of *DNMT3a*-prom-luc activity by MTA1 overexpression as compared to the control MCF-7 cells (Fig. 4A). Similarly, we observed an increase in *DNMT3a-*prom-luc activity when MTA1 is silenced in MCF-7 cells (Fig. 4B). Consistent with these results, the basal *DNMT3a*-prom-luc activity increases by about 4-fold in MTA1-KO MEF as compared to the wild-type MEF (Fig. 4C).

To gain an insight of the regulation of the *DNMT3a* transcription by MTA1, we next mapped the recruitment of the MTA1/NuRD complex onto the *DNMT3a* chromatin by promoter walk-based ChIP analysis. Results showed that MTA1 is recruited onto the *DNMT3a* promoter region R1 (−402 to −220 bp) but not to regions 2 (−854 to −687 bp) and 3 (−1501 to −1142 bp) (Fig. 4D). Because MTA1 interacts with the target chromatin as a part of HDAC-containing NuRD complexes, we performed ChIP analyses using an anti-HDAC1-Ab or anti-HDAC2-Ab and found that HDAC1 but not HDAC2, interact with all regions of the *DNMT3a* chromatin (Fig. 4D). However, results of sequential double ChIP studies involving MTA1 ChIP followed by HDAC1 ChIP indicated that MTA1/HDAC1 complex only interacts with the region 1 of the *DNMT3a* promoter (Fig. 4D).

Because MTA1 does not bind to the DNA directly but utilizes transcriptional factors, we next analyzed the region 1 (−402 to −220 bp) of the *DNMT3a* promoter for transcription factors binding motifs using ALLGEN PROMO and found one binding sites for the YY1 transcription factor. Previous studies have shown that MTA1 inhibits the expression of *PTEN* tumor suppressor through YY1 transcription factor^20^. To assess the relevance of YY1 in the action of MTA1, we performed either single ChIP or sequential double ChIPs involving MTA1-Ab ChIP followed by YY1-Ab ChIPs onto the *DNMT3a* chromatin and compared with the binding patterns of individual molecules (Fig. 4E). Results indicated that MTA1 along with YY1 is recruited onto the *DNMT3a* promoter along with HDAC1 as all three molecules are co-recruited on the region 1 of the *DNMT3a* promoter. To further understand the details of MTA1 regulation of DNMT3a expression, we used *DNMT3a*-pro-Luc system. We found that YY1 overexpression decreases the levels of *DNMT3a* promoter activity when compared to control vectors in MCF-7 cells. Together, these results suggest that the noticed repression of *DNMT3a*-transcription by the MTA1-NuRD complex may be mediated through YY1 transcriptional factor (Fig. 4F).

### MTA1 modulation of DNMT3a targets

**Figure 5:**
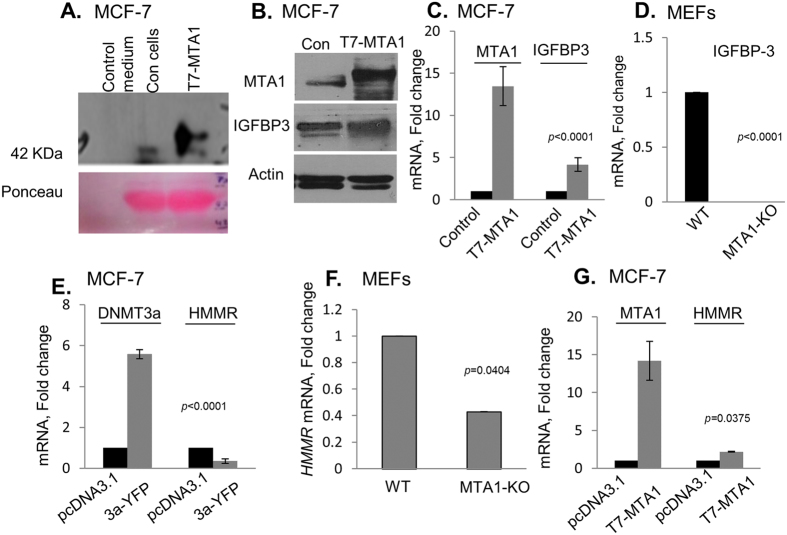
MTA1 modulation of DNMT3a target genes. (**A**) Western blot showing secreted IGFBP-3 in the conditioned medium from MCF-7 and MCF-7/T7-MTA1 cells; ponceau staining was used to show the equal loading of protein. (**B**) Western blot showing the IGFBP3 levels from MCF-7 and MCF-7/T7-MTA1 cells. β-actin is shown as the loading control. (**C**) qPCR analysis of IGFBP-3 in MTA1 overexpressed MCF-7 cells as compared with the vector control. Results were represented in fold change normalized to mRNA levels of 18 S rRNA. (**D**) qPCR analysis of IGFBP-3 in MTA1^+/+^ and MEF MTA1^−/−^ MEFs. (**E**) qPCR showing the levels of DNMT3a and HMMR mRNAs in MCF-7 and in MCF-7 cells overexpressing DNMT3a-YFP or pcDNA3.1 as a vector control. (**F**) qPCR analysis of HMMR mRNAs in wild-type and MEF MTA1^−/−^ MEFs. Results are presented as fold change after normalizing with β-actin. (**G**) qPCR analysis of MTA1 and HMMR mRNAs in MCF-7 and in MTA1 overexpressing MCF-7 cells. Results are presented as fold change after normalizing with β-actin.

To understand the potential implication of MTA1 regulation of DNMT3a expression on downstream targets, we selected IGFBP3, a previously characterized repressed target gene by DNMT3a^15^. The IGFBP3, a secreted factor which could act both as an oncogene or a tumor suppressor in a context dependent manner^21^. We first analyzed the level of secreted IGFBP3 and cellular IGFBP3 in MCF-7 and MTA1 overexpressing breast cancer cells. We noticed an increased amount of secreted IGFBP3 in the concentrated conditioned medium from MTA1-overexpressing cells as compared to control cells (Fig. 5A). In addition, there was a modest increased expression in the levels of cellular IGFBP3 in MCF-7 overexpressing cells (Fig. 5B). Further, MTA1-overexpressed MCF-7 cells showed increased levels of IGFBP3 mRNA (Fig. 5C) when compared to the control cells. We also observed a significant decrease in the levels of IGFBP-3 mRNA in MTA1-KO-MEF as compared to wild-type MEF (Fig. 5D).

To demonstrate that the noted regulation of IGFBP-3 by MTA1-DNMT3a axis may be reflected for other target genes, we next evaluated the effect of MTA1 status on the levels of DNMT3a target gene(s). An earlier microarray data has identified Hyaluronan-Mediated Motility Receptor (HMMR) as a target of DNMT3a as the levels of HMMR were shown to be upregulated by selective knock down of DNMT3a^9^. In this context, we noticed that MTA1 overexpression in MCF-7 cells also results in an increased levels (*p *<* 0.0375*) of HMMR mRNA as compared to the levels in cells expressing the control vector (Fig. 5E), while genetic depletion of MTA1 in MTA1-KO-MEFs leads to a reduced HMMR mRNA expression as compared to the levels of HMMR mRNA in the control wild-type MEF (*p *<* 0.04*) (Fig. 5F), presumably due to increased expression of DNMT3a by MTA1 depletion (Fig. 2). Consistent with these observations, DNMT3a overexpression in MCF-7 cells leads to a significant reduction in the expression of HMMR mRNA (Fig. 5G). These results suggest that MTA1-DNMT3a axis may regulate the expression of a sub-set of DNMT3a-regulated genes. Since the expression of target genes is regulated by a variety of cis- and trans-regulatory mechanism and not always regulated by a linear pathway or pathways, it is conceivable that there would be sub-sets of genes regulated by MTA1 or DNMT3a, independent of each other. Therefore, to fully understand the global status of MTA1-regulated but DNMT3a-dependent genes, it would be important in future to perform genome-wide RNA-sequencing studies under conditions of MTA1 or DNMT3a knockdown using DNMT3a-KO or MTA1-KO MEFs, respectively. In addition, such future studies may include MTA1 knockdown with or without DNMT3a in breast cancer cells.

### MTA1 stimulates IGFBP3 expression

**Figure 6:**
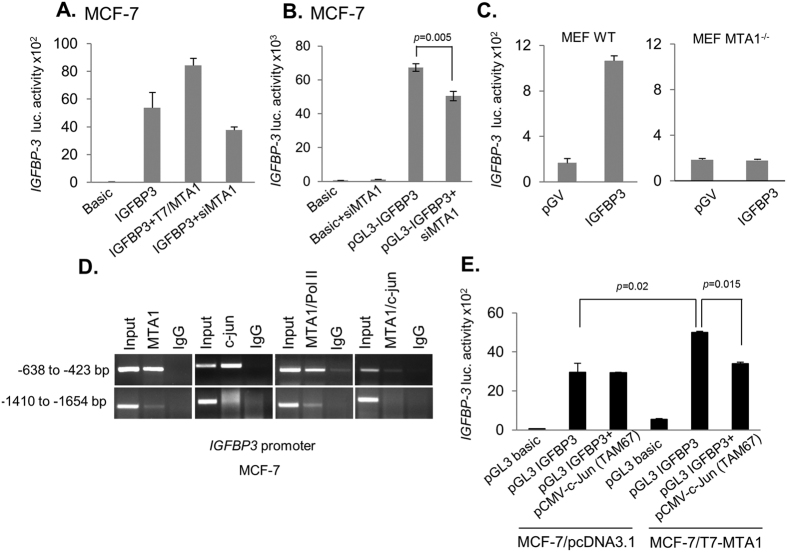
MTA1 regulates IGFBP-3 expression. (**A**) *IGFBP-3* promoter activity in MCF-7 and in MTA1-overexpressed MCF-7 cells. (**B**) MCF-7 cells were transfected with pGL3-IGFBP3 along with siMTA1 and *IGFBP-3*-promo-Luc activity was measured. (**C**) *IGFBP-3* promoter activity in MTA1^+/+^ and MTA1^−/−^ MEFs. (**D**) ChIP analysis with anti-MTA1 showing the recruitment of MTA1 or c-Jun or MTA1/c-Jun or MTA1/PolII complexes onto various regions of the *IGFBP-3* promoter. (**E**) MCF-7 cells were transiently transfected with pGL3-IGFBP-3 reporter construct with vector alone or along with pCMV-TAM67-c-jun along with pGL3-basic. Renilla luciferase activity was normalized and the effect of c-jun on *IGFBP3*-promoter activity was measured. Results were presented as relative luciferase activity and the values represent the mean of ± s.d. from three independent transfection experiments.

Consistent with these findings, MTA1 overexpression increases the level of *IGFBP3*-promoter activity as compared to the control cells (Fig. 6A) and MTA1-silencing leads to a decreased *IGFBP3*-promoter activity as compared to the levels in the control cells (Fig. 6B). These results revealed a potential coactivator function of MTA1 upon IGFBP3 expression. Consistent with these findings, we noticed an elevated *IGFBP3*-promoter activity in wild-type MEFs as compared to MTA1-KO MEFs (Fig. 6C). Because MTA1 also acts as a coactivator of its target genes via interacting with transcription factors such as c-Jun transcription factor^22^, we also explored the possibility of recruitment of the MTA1/c-Jun coactivator complex onto *IGFBP3* promoter chromatin using ChIP assays. We found that indeed, MTA1 or c-Jun or MTA1/c-Jun are effectively recruited onto the region 1 (−638 to −423 bp) of the *IGFBP3*-promoter (Fig. 6D). To further demonstrate a coactivator function of c-Jun upon *IGFBP3*-transcription, we next examined the effect of inactivating the endogenous c-Jun by a dominant-negative expression vector (pCMV-c-Jun(TAM67)^23^ on the *IGFBP3*-promoter activity^24^. We noticed that MTA1 overexpression is accompanied by an increased simulation of the *IGFBP3* promoter activity as compared to the levels in the control cells. Such an increased activity could be partly, but not fully, compromised by inactivating c-Jun functionality (Fig. 6E). As coactivator functions of MTA1 upon IGFBP3 cannot be fully explained by c-Jun transcription factor alone, and that future studies on the nature of additional pathways might be of interest. Together these observations suggest that MTA1 may act as a cofactor of *IGFBP3* transcription via recruiting c-Jun transcription factor. Because previous studies have shown that DNMT3a represses IGFBP3 expression^15^,^16^, our present findings raise the possibility that MTA1 could regulate the expression of IGFBP3 in both DNMT3a-dependent and -independent manner and that in-principal, MTA1 could regulate the expression of IGFBP3 through more than one pathways.

### Status of MTA1, DNMT3a, and IGFBP3 in Breast Cancer

**Figure 7:**
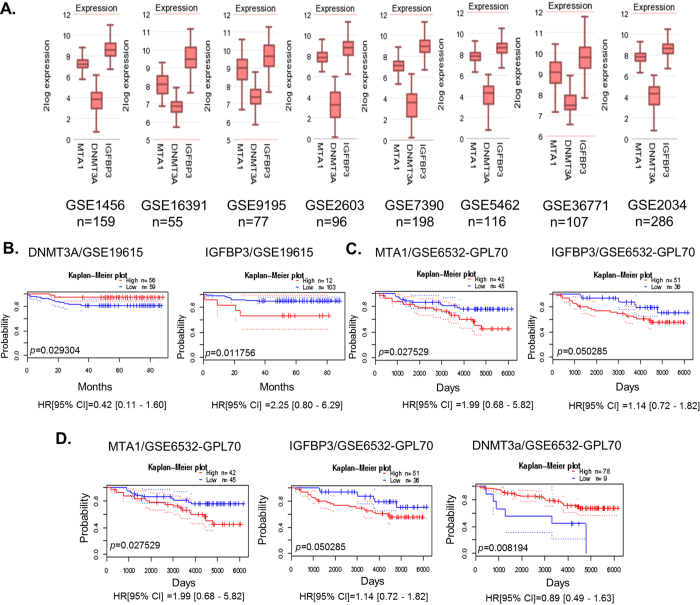
Clinical significance of MTA1/DNMT3a/IGFBP-3 axis in breast cancer. (**A**) Three-way correlation of MTA1 and DNMT3a mRNAs in eight breast cancer datasets as analyzed using R2 Gene view platform. The values are presented on a log2 scale. (**B**) Kaplan-Meier plot of distant metastasis free survival of 115 breast cancer patients stratified by high (red) and low (blue) DNMT3a levels (high: *n* = 56, low: *n* = 59; *p* = 0.029304) and IGFBP-3 levels (high: *n* = 12, low: *n* = 103; *p* = 0.011756) in the dataset GSE19615. (**C**) Kaplan-Meier plot of relapse free survival of 87 breast cancer patients stratified by high (red) and low (blue) MTA1 levels (high: *n* = 42, low: *n* = 45; *p* = 0.027529) and IGFBP3 levels (high: *n* = 51, low: *n* = 36; *p *= 0.050285) in the dataset GSE6532-GPL60. (**D**) Kaplan-Meier plot of distant metastasis free survival of 87 breast cancer patients stratified by high (red) and low (blue) MTA1 levels (high: *n* = 42, low: *n* = 45; *p* = 0.027529) and IGFBP3 levels (high: *n* = 51, low: *n* = 36; *p *= 0.050285) along with the DNMT3a levels (high: *n* = 78, low: *n* = 9; *p* = 0.008194) in the dataset GSE6532-GPL60 analyzed through PrognoScan database, dotted lines indicate 95% confidence intervals for each group.

To understand the physiological relevance of MTA1 stimulation of IGFBP3 expression in cancer, we examined the status of MTA1 and IGFBP3 in context of DNMT3a expression in human tumors. We first searched for possible correlations between MTA1, DNMT3a, and IGFBP3 using eight breast cancer datasets (Fig. 7A). We found that low levels of DNMT3a correlate well with a high level of IGFBP3 or MTA1 expression in the majority of the data sets. To assess the clinical significance of MTA1/DNMT3a/IGFBP3 axis in breast cancer, we performed a two-way correlation involving low levels of DNMT3a and high levels of IGFBP3 associated with a poor distant metastasis-free survival (Fig. 7B). High levels of MTA1 and high levels of IGFBP3 associates with a poor relapse-free (Fig. 7C) and metastasis-free (Fig. S2) survival of breast cancer patients. Interestingly, high levels of MTA1, low levels of DNMT3a, and high levels of IGFBP3 correlate with a poor distant metastasis-free survival (Fig. 7D) as well as relapse-free survival (Fig. S3). Overall, the three-way analysis of MTA1, DNMT3a and IGFBP3 provided us with some interesting clue about the clinical significance of MTA1/DNMT3a/IGFBP3 axis, and highlighted a potential role of MTA1 regulation of IGFBP3 in the context of MTA1 repression of DNMT3a expression. In future studies, the authors anticipate taking these studies to genetic murine models of breast cancer progression.

## Discussion

Recent evidence suggest that DNMT3a, apart from its role in DNA methylation, also acts as an oncogene in melanoma, pancreatic cancer, gastric cancer and hepatocellular carcinoma^25^,^26^,^27^,^28^, while it acts as a tumor suppressor in lung cancer^12^. However, the expression of DNMT3a in breast cancer remains unclear. Earlier immunohistochemistry staining of DNMTs in primary and metastatic breast cancer tissues showed a substantial lower level of DNMT3a in metastatic tissue as compared to the primary stage^29^. In this context, using data-mining of a variety of available datasets, we recognized that the levels of MTA1 inversely correlate with DNMT3a status in breast cancer (as well as in other cancer-types)^30^,^31^. In general, as compared to the downstream targets of DNMT3a, there are very few studies focusing on the nature of upstream regulators of DNMT3a^32^. For example, the UHRF family of E3 ligases, have been shown to inhibit the expression of DNMT3a^33^. Here we show that MTA1 chromatin remodeling factor negatively regulates the transcription of *DNMT3a* via utilizing a HDAC1/YY1 corepressor complex. Interestingly, we noted some degree of relative preference for HDAC1 over HDAC2 in the MTA1/HDAC1/YY1 corepressor complex, consistent with the idea of non-reductant nature of the same NuRD complexes. The regulation of DNMT3a by MTA1 may be clinically relevant as low and high levels of DNMT3a and MTA1, respectively, may be associated with a poor survival of patients not only with breast cancer but also in other cancers. Recently, DNMT3a has been shown to also serve as an independent prognostic marker in lung cancer where weak DNMT3a expression may result in a poor outcome^25^. Low levels of DNMT3a in metastatic specimens support the notion that MTA1 upregulation may drive the process of metastasis. It is possible that the noted DNMT3a downregulation could be an effector event of MTA1 overexpression during breast cancer progression. Further it is possible that MTA1-DNMT3a axis may be associated with a potential hypo-methylation of certain, yet-to-be identified, target genes in cancer. Down regulation of DNMT3a has been also shown to be associated with a poor overall survival in AML patients^34^. Similarly, DNMT3a deletion in lung cancer promotes cancer progression^12^. Apart from being involved in the cancer progression, low levels of DNMT3a could result in an unscheduled activation of *EPAS1* gene which contributes to cell survival under extreme hypoxic conditions^10^. In addition to cancer progression, DNMT3a may be a negative regulator of tyrosine hydroxylase (TH1) as paraventricular nucleus of the hypothalamus (PVH) in Sim1-specific Dnmt3a deletion mice exhibits an elevated TH level^35^,^36^. In this context, it is interesting to note that MTA1 - also an repressor of DNMT3a (this study), acts as a coactivator of *TH* transcription in neuronal cells^35^.

Our observation that MTA1 acts as a coactivator to stimulate the *IGFBP3* through c-Jun/PolII co-activator complex is in-line with a dual master coregulatory nature of MTA1^21^. Interesting, in addition to MTA1, IGFBP3 is also overexpressed in many other human cancers [13; 36; 37]. However, results from IGFBP3^−/−^ knockout mice shows an accelerated tumor growth as compared to the wild type mice, suggesting anti-oncogenic role of IGFBP3 in this specific experimental model^36^. Thus, IGFBP3 may contribute to its growth inhibitory function in both insulin-like growth factor-dependent and -independent manner. In general, an increased status of IGFBP3 correlates with a poor prognosis in cancer, at-large, including in breast cancer. In triple-negative breast cancers, the levels of IGFBP3 are arguably high and results in a poor prognosis as estrogen represses IGFBP3.

Because IGFBP3 is an established target of DNMT3a^15^ and the fact MTA1 represses DNMT3a (this study), our findings of MTA1 regulation of IGFBP3 suggest an alternative, indirect mechanism of MTA1-mediated upregulation of IGFBP3 via inhibiting the expression of DNMT3a, a repressor of IGFBP3. Studies from microarray data reveal that MTA1 may be an upstream regulator of IGFBP3 as well as DNMT3a (Fig. S4). In the past, DNMT3a has been shown to epigenetically regulate the levels of IGFBP3 in cervical cancer by CUL4B;^15^ DNMT3a deprivation in liver cancer results in an increased IGFBP3 expression^16^. In the same vein, we also noticed an increased expression of IGFBP3 in DNMT3a^−/−^ mice as compared to DNMT3a^+/+^ mice in a public microarray dataset GSE32487^12^ (Fig. S5). However, we also present evidence of DNMT3a-independent regulation of IGFBP3 by MTA1. Overall, results presented here show that the high levels of IGFBP3, low levels of DNMT3a, and high levels of MTA1 may result in poor prognosis of cancer patients and that the expression of IGFBP3 by MTA1 could be regulated by direct or indirect mechanisms.

## Conclusion

**Figure 8:**
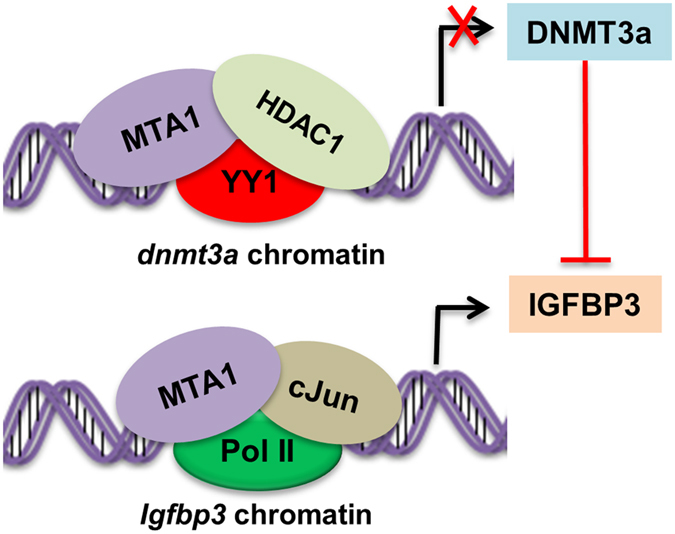
Schematic model showing the repression of DNMT3a by MTA1 through MTA1/HDAC1/YY1 corepressor complex and transcriptional upregulation of IGFBP3 via MTA1/c-jun/Pol II coactivator complex.

We have found that MTA1 chromatin regulator regulates the expression of DNMT3a via MTA1-containing MTA1/HDAC1/YY1 corepressor complex. In addition to its corepressor functions, MTA1 was found to stimulate the expression of IGFBP3 - via recruiting the MTA1/PolII/c-Jun coactivator complex onto the *IGFBP3* (Fig. 8). Further this study highlights the clinical significance of MTA1, DNMT3a and IGFBP3 in determining an overall poor prognosis of cancer patients.

## Materials and Methods

### Cell Culture and Reagents

MCF-7, SKBR-3, MCF-7/T7-MTA1, MEF WT, MEF MTA1^−/−^, HCT-116 cells were used in the study. RPMI 1640 (Invitrogen, CA) supplemented with 10% FBS and 1X antibiotic/antimycotic (15240062, Gibco, CA) was used to maintain the cells in a humidified 5% CO_2_ at 37 °C. Antibodies used were MTA1 (#5447, Cell Signaling Technology), DNMT3a (ab188470, Abcam), IGFBP3 (sc-9028, Santa Cruz), HDAC1 (ab46985, Abcam), HDAC-2 (sc-7899, Santa Cruz), Vinculin (sc-73614, Santa Cruz), RNA Polymerase II (A300-654A, Bethyl labs), Dnmt3a (sc-20703, Santa Cruz), YY1 (#2185, Cell Signaling Technology) and β-Actin (sc-47778, Santa Cruz). Secondary antibodies conjugated to Horseradish peroxidase were obtained from Sigma-Aldrich. Chemiluminescence detection reagents were obtained from Millipore (WBKLS0100). All other reagents were purchased from Sigma-Aldrich, if not mentioned.

### Western Blotting and Immunoprecipitation

Phospholysis buffer containing 50 mM Tris-HCl, pH 7.4, 1% Nonidet P-40, 150 mM NaCl, 1 mM EDTA, 0.25% sodium deoxycholate with 1X protease inhibitor cocktail (P8340, Sigma) was used for preparing protein lysates and their protein concentrations were determined. Protein lysates were then resolved using SDS-PAGE. Resolved proteins were transferred to PVDF membranes (GE Amersham Biosciences) and blocked with 5% milk powder in TBST for 75 minutes and then incubated with indicated primary antibodies for overnight at 4 °C. After washing the primary antibodies, blots were incubated with secondary antibodies for 90 minutes at room temperature. Detection was performed using the ECL reagent or by DAB after sufficient washing.

### siRNA Transfection

Specific siRNA for MTA1 (sc-35981) was purchased from Santa Cruz and used at a final concentration of 100 nM. Control siRNA (D-001810-10) was purchased from GE Dharmacon. Lipofectamine RNAiMAX (Invitrogen) was used for siRNA Transfections carried out in 6-well plate using with Opti-MEM (Invitrogen) according to the manufacturer’s protocol. Plasmids indicated were transiently transfected into the cells in 6-well plate using Lipofectamine LTX (Invitrogen) with Opti-MEM in accordance with the manufacturer’s instructions.

### Real Time qPCR

TRIzol reagent (Invitrogen, Carlsbad, CA) was used for isolating total RNA from the cells. Briefly, cells were lysed with TRIzol and incubated for 5 minutes at room temperature. 250 μl of chloroform was added and shaken vigorously for 1 minute and incubated for 5 minutes at room temperature. After centrifugation at 10000 rpm for 5 minutes, aqueous layer is collected and RNA is precipitated by adding isopropanol. After centrifugation at 14000 rpm for 30 minutes, RNA pellet is washed with 80% ethanol and air dried. Finally resuspended in nuclease free water. RNA concentration was estimated using Nanodrop and two micrograms of extracted RNA was converted to cDNA. The resultant cDNA was subjected to qPCR by using SYBR^®^ Premix Ex Taq™ II Tli RNase H Plus (Takara) using StepOnePlus Real Time PCR system (Applied Biosystem, CA). The values for specific genes were normalized using β-actin or 18 S rRNA as housekeeping control. The primers used for qPCR are provided in the supplementary information.

### Chromatin Immunoprecipitation

Chromatin immunoprecipitation assays were done using Simple ChIP kit (Cell Signaling) as per protocol mentioned in the kit. Briefly, approximately 10^6^ cells were crosslinked by treating with formaldehyde to a final concentration of 1% for 7 min at 37 °C and neutralized with glycine (final concentration 125 mM) for 7 minutes. The cross linked cells were washed with ice cold phosphate-buffered saline (PBS) with protease inhibitors cocktail. Cells were lysed by sonication (12 cycles, 30 sec pulse) and immunoprecipitated with specific antibodies for overnight at 4 °C. The immunoprecipitates were washed and eluted using 100 mM NAHCO3 in 1% SDS at 37 °C for 1 hr. De-crosslinking was done using NaCl and after proteinase K digestion, DNA purified using phenol-chloroform extraction was subjected to PCR. For double ChIP, the eluted samples were five-fold diluted and subjected to subsequent antibodies. PCR primers for ChIP assays were provided in the supplementary information.

### Promoter Luciferase Assay

Dual luciferase assay was carried out according to the manufacturer’s protocol (Promega, Cat. no. E1910) using a Luminometer (TD20/20, Promega). Briefly, cells were transfected in a 24 well plate with the mentioned plasmids, harvested after 48 h of transfection using 1X passive lysis buffer for 20 min followed by centrifugation at 12000 rpm for 5 min and the supernatant was used to carry out the assay. Renilla luciferase was used to normalize the difference in transfection efficiency between samples. All the values were expressed as the mean ± standard deviation of at least three independent experiments carried out in triplicates.

### Data Mining

Gene expression of MTA1, DNMT3a and IGFBP3 were downloaded from R2: Genomics Analysis and Visualization Platform (http://r2.amc.nl) for breast cancer (GSE2034, GSE1456, GSE36771, GSE16391) and colon cancer datasets (GSE4183, GSE35896). The numbers of samples used in the datasets are indicated. Oncomine was used to analyse the gene expression of DNMT3a and MTA1 both in normal and cancer tissues. cBioPortal 37,38 database was used to extract the expression of MTA1 and DNMT3a in TCGA and Cancer Cell Line Encyclopedia (from all complete tumors) (http://www.cbioportal.org/). Gene expression of MTA1 and DNMT3a were also extracted from CAncerREsource 2 database (http://data-analysis.charite.de/care/). PrognoScan database was used for the Kaplan-Meier survival analysis (http://www.abren.net/PrognoScan/). Series matrix files for the datasets (GSE25072, GSE32487) were downloaded from Gene Expression Omnibus Database for the indicated datasets and gene expression of DNMT3a and IGFBP3 were analyzed. Results were represented in fold change. (Probe used for dataset GSE25072: 6785865/IGFBP3, 6792945/DNMT3a; Probe used for dataset GSE32487: A_51_P228472/IGFBP3).

### Data Analysis

Results are expressed as the mean ± SD. Statistical significance was evaluated with a two-tailed, unpaired Student’s *t*-test.

## Additional Information

**How to cite this article**: Deivendran, S. *et al*. Metastasis-associated protein 1 is an upstream regulator of *DNMT3a* and stimulator of insulin-growth factor binding protein-3 in breast cancer. *Sci. Rep*. **7**, 44225; doi: 10.1038/srep44225 (2017).

**Publisher's note:** Springer Nature remains neutral with regard to jurisdictional claims in published maps and institutional affiliations.

## Supplementary Material

Supplementary Information
