# Supplementary material for: Metastasis-associated protein 1 is an upstream regulator of DNMT3a and stimulator of insulin-growth factor binding protein-3 in breast cancer
Source: Sci Rep. 2017 Apr 10;7:44225. doi: 10.1038/srep44225 (PMC5385551; doi:10.1038/srep44225)
Supplement: Supplementary Information [file srep44225-s1.pdf]

Metastasis-associated protein 1 is an upstream regulator of *DNMT3a* and stimulator of insulin-growth factor binding protein-3 in breast cancer

S. Deivendran[1], Hezlin Marzook[1], T.R. Santhoshkumar[1], Rakesh Kumar[1,2*] and M. Radhakrishna Pillai[1*]

Supplementary information:

Primers:

Quantitative PCR:

hMTA1

FP: CTCTGCGCATCTTGTTGGACATA

RP: TCAGCTTCGTCGTGTGCAGATAG

hβ-ACTIN

FP: AGACTTCGAGCAGGAGATG

RP: CTTGATCTTCATGGTGCTAGG

18s rRNA

FP: GGCCCTGTAATTGGAATGAGTC

RP: CCAAGATCCAACTACGAGCTT

hDNMT3A

1. FP: CCTGTGGGAGCCTCAATGTTA
   RP: CTTGCAGTTTTGGCACATTCC
2. FP: CCAACATCGAATCCATGAAA
   RP: CTTGCGCTTGCTGATGTAGT

IGFBP3

FP: CAAGCGGGAGACAGAATATGG

RP: CAGGTGATTCAGTGTGTCTTCCA

HMMR

FP: AGGCAATACAAACTGTTACC

RP:ATCCTTCAACAGGTTTCTCAG

DNMT3b

FP: CGAATTTTACCACCTGCTGAATT

RP: AGAACGGCCGGTCATCAC

mDNMT3a

FP:ACTTGGAGAAGCGGAGTG

RP:CTGTTCTTTGCCCTCTCCTG

mMTA1

FP:GGCCAAAGTGGTGTGTTTCT

RP:GAGACTCCAACTGCCGAGAC

mACTIN

FP: TGTTACCAACTGGGACGACA

RP: CTGGGTCATCTTTTCACGGT

mHMMR

FP:CAAGAAATTAGCTGTGCATC

RP: GCATTTAACAGTCAGGTCTTC

mIGFBP3

FP:CTGAATCATCTGAAGTTCCTC

RP:GGCACTGCTTCTTCTTATAG

ChIP primers

DNMT3a

Region 1:

FP: AGGAACCTAGAGCCCTGAGC

RP: GTGAGTTCCCCGTACCTTGA

Region 2

FP: AGGAAAACCTTCCCGTCTGT

RP: GTTGGTGGTGGGACTCACTT

Region 3

FP: ATGTGGGGTGATTTCAGAGC

RP: CATATAACACGGCCCCAAAC

IGFBP3

Region 1

FP: GCGTTGAGAAGTAAGCCTGG

RP: GCATTCGTGTGTACCTCGTG

Region 2

FP: TGAGCACTGTGGGAGAAGAG

RP: TCTCTCGTCCTCTCCCCTTT

Supplementary Figure Legends

Supplementary Figure 1: (S1A) Levels of MTA1 and DNMT3a inversely correlate in breast cancer. Oncomine Cancer Profiling Database was used to analyze the MTA1 and DNMT3a mRNAs in normal (n=6) and in breast carcinoma (n=53). (S1B) Representative datasets showing the prognostic value of MTA1 and DNMT3a analyzed through PrognoScan, where high levels of MTA1 ($p<0.001$) [panel 1] and low levels of DNMT3a ($p<0.05$) [panel 2] correlates with the poor overall survival in cancers

Supplementary Figure 2. Kaplan-Meier plot of distant metastasis free survival of 198 breast cancer patients stratified by high (red) and low (blue) MTA1 levels (high: *n*=176, low: *n*=22; *p*=0.076940) and IGFBP3 levels (high: *n*=85, low: *n*=113; *p*=0.009026) in GSE7390 data analyzed through PrognoScan database, dotted lines indicates 95% confidence intervals for each group.

Supplementary Figure 3: High levels of MTA1, low levels of DNMT3a and high levels of IGFBP-3 correlates with the poor relapse free survival in breast cancer patients. Kaplan-Meier plot of relapse free survival of 77 breast cancer patients stratified by high (red) and low (blue) MTA1 levels (high: *n*=30, low: *n*=47; *p*=0.014942), DNMT3a levels (high: *n*=45, low: *n*=32; *p*=0.023859) and IGFBP3 levels (high: *n*=8, low: *n*=69; *p*=0.002132) in the dataset GSE9195 analyzed through PrognoScan database, dotted lines indicates 95% confidence intervals for each group.

Supplementary Figure 4. Microarray dataset (GSE25092) showing the levels of DNMT3a and IGFBP3 mRNAs in the wild-type (WT) and MTA1$^{-/-}$ MEFs.

Supplementary Figure 5. Microarray dataset (GSE32487) showing changes in the levels of IGFBP3 and DNMT3a mRNAs in the wild-type (WT) and in DNMT3a$^{-/-}$mice.

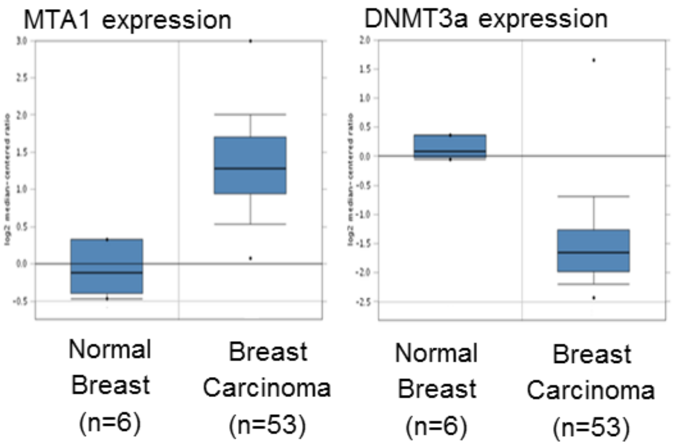

p-value= 2.09E-7
Fold change =2.749

p-value= 5.41E-16
Fold change =-3.158

Finak et al., 2008

Figure S1A

High MTA1 expression Vs patient survival

CANCER TYPE	DATASET	P VALUE	HR (95% CI)
Ovarian Cancer	DUKE-OC	0.001048	1.25 [0.92 - 1.70]
Brain Cancer	GSE7696	0.000737	2.07[0.41 - 10.35]
Lung Cancer	GSE13213	0.000175	0.48 [0.31 - 0.75]
Bladder Cancer	GSE5287	0.000024	6.98(2.11-23.15)
Colon Cancer	GSE17536	0.001103	2.12(1.22-3.71)

Low DNMT3a expression Vs patient survival

CANCER TYPE	DATASET	P VALUE	HR (95% CI)
Ovarian Cancer	GSE9891	0.056	0.90 [0.69 - 1.18]
Lung Cancer	GSE13213	0.017	0.80(0.54-1.19)
Lung Cancer	Jacob-00182UM	0.05	0.78 (0.60-1.00)
Bladder Cancer	GSE13507	0.037	0.69(0.14-0.34)
Blood Cancer	GSE8970	0.035437	0.82(0.59-1.14)

Figure S1B

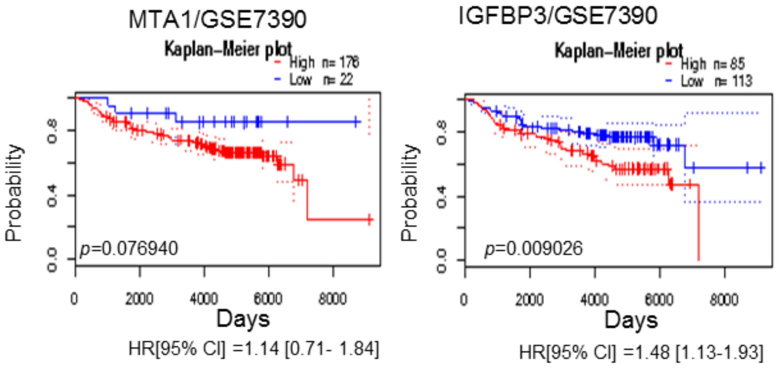

Figure S2

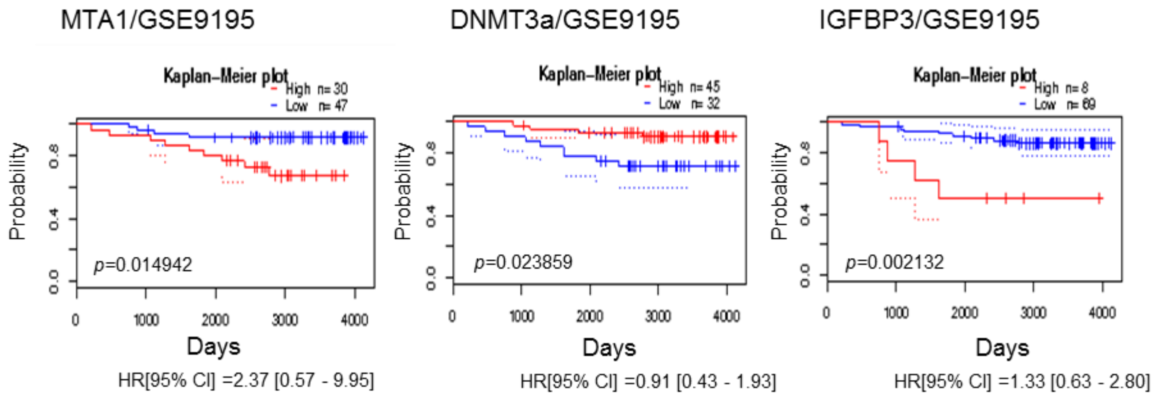

Figure S3

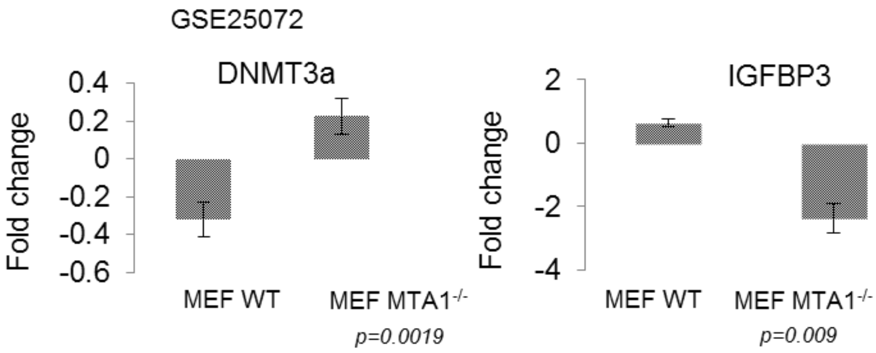

Figure S4

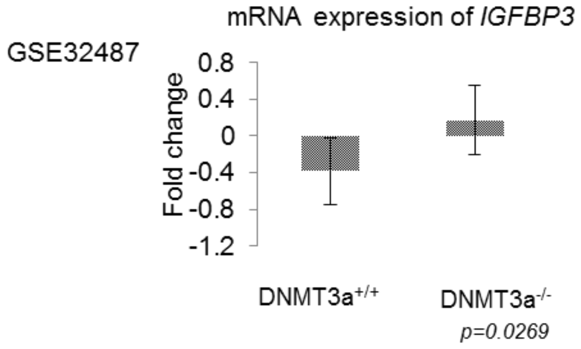

Figure S5
